# Lung function, pharmacokinetics, and tolerability of inhaled indacaterol maleate and acetate in asthma patients

**DOI:** 10.1186/s12931-020-01501-1

**Published:** 2020-09-23

**Authors:** David Miller, Soniya Vaidya, Juergen Jauernig, Brian Ethell, Kristina Wagner, Rajkumar Radhakrishnan, Hanns-Christian Tillmann

**Affiliations:** 1 Northeast Medical Research Associates Inc., North Dartmouth, MA USA; 2 Axcella Health, Cambridge, MA USA; 3 Novartis Pharma AG, Basel, Switzerland; 4 Novartis Institutes for Biomedical Research, Cambridge, MA USA; 5 Novartis Healthcare Pvt. Ltd., Hyderabad, India; 6 Novartis Institutes for Biomedical Research, Basel, Switzerland

**Keywords:** Asthma, Pharmacokinetic, Pharmacodynamics, Efficacy, LABA, Randomised control trial

## Abstract

**Background:**

Indacaterol maleate delivered with the Breezhaler® inhalation device is a long-acting β_2_-agonist approved for chronic obstructive pulmonary disease. In the development of a once daily, inhaled fixed dose combination (FDC) of indacaterol, glycopyrronium bromide (a long-acting muscarinic antagonist), and mometasone furoate (an inhaled corticosteroid [ICS]) for the treatment of patients with asthma, the acetate salt of indacaterol is used instead of the maleate salt. Here, we investigated the lung function, pharmacokinetics (PK) and safety of indacaterol maleate 150 μg once daily (o.d.) and indacaterol acetate 150 μg o.d. in comparison with placebo.

**Methods:**

This was a randomised, double-blind, three-period crossover study (ClinicalTrials.gov identifier, NCT03257995) in patients with asthma on background ICS therapy. Patients with percent predicted pre-bronchodilator forced expiratory volume per second (FEV_1_) ≥50% and ≤ 90% were included in the study. Patients received indacaterol maleate 150 μg o.d., indacaterol acetate 150 μg o.d., or placebo on top of stable background ICS in randomised sequence. Trough FEV_1_ was assessed after 14 days of treatment. PK of indacaterol salts were assessed at steady state after 14 days of treatment; peak expiratory flow (PEF) rate and rescue medication use were collected with a combined PEF-meter/electronic diary throughout the study.

**Results:**

Of the 54 adult patients (median age of 48 years), 51 patients completed the study. Both indacaterol salts demonstrated statistically significant improvements in trough FEV_1_ of 186 mL (maleate) and 146 mL (acetate) compared with placebo (both *P* < 0.001). FEV_1_ AUC_0-4h_ improved by 248 mL (maleate) and 245 mL (acetate), and PEF by 33 L/min (maleate) and 30.8 L/min (acetate) versus placebo. Systemic exposure of indacaterol (AUC_0-24h,ss_ and C_max,ss_ on Day 14) was comparable after administration of both salt forms. Both salt forms demonstrated a good safety profile and were well tolerated, with a difference in the reporting frequency of AEs of coughing (maleate, 23.5%; acetate, 0%).

**Conclusions:**

In patients with asthma, indacaterol maleate and acetate elicited comparable and significant improvements in lung function compared with placebo and achieved comparable systemic exposure. Both indacaterol salts were safe and well tolerated.

**Trial registration:**

ClinicalTrials.gov NCT03257995 June 06, 2017

## Introduction

Asthma is a chronic inflammatory disorder of the airways, associated with airway hyper-responsiveness that leads to recurrent episodes of wheezing, breathlessness, chest tightness, and coughing [1, 2]. The focus of asthma management strategies is symptom control, lung function improvement, and reduction in the risk of asthma-related exacerbations [1].

In patients with persistent symptoms and exacerbations, the Global Initiative for Asthma (GINA) 2019 report recommends the use of high-dose inhaled corticosteroids (ICS) and/or ICS plus long-acting β_2_-agonists (ICS/LABA) [1]. In patients who remain uncontrolled on medium- or high-dose ICS/LABA, add-on treatment with long-acting muscarinic antagonist (LAMA), tiotropium, is a proposed treatment option [1].

Indacaterol maleate was the first LABA approved for use in the EU (2009) as a once-daily (o.d.) maintenance monotherapy in patients with chronic obstructive pulmonary disease (COPD) as Onbrez® Breezhaler® [3]. In 2013, the combination therapy of indacaterol maleate and the LAMA glycopyrronium was approved in the EU and Japan as an once-daily dual bronchodilator therapy in COPD (Ultibro® Breezhaler® [4, 5]). Indacaterol maleate shows a rapid onset of action within minutes of administration and provides 24 h bronchodilation in patients with COPD [6]. Since its approval, the efficacy and safety of indacaterol in patients with COPD has been established through numerous randomised controlled trials and real-world evidence studies [6–9].

The INVOLVE study which involved COPD patients, which assessed the long-term (52-week) efficacy and safety of indacaterol maleate (150 μg o.d., delivered with the Breezhaler® device) showed a significant improvement in trough forced expiratory volume in 1 s (FEV_1_) over formoterol (12 μg twice daily [b.i.d.]) [7, 10]. Several studies have demonstrated that indacaterol maleate is highly potent and has a good safety profile in patients with asthma and COPD [11–15].

In Phase III clinical studies of indacaterol maleate in COPD patients, healthcare providers observed during clinic visits that some patients experienced a sporadic cough that occurred usually within 15 s following inhalation and typically lasted for 5 s (about 10 s in current smokers). Indacaterol acetate was considered to potentially elicit less coughing [16]. Indacaterol acetate, an alternative salt form of indacaterol maleate, has been developed as part of two different once-daily inhaled fixed dose combinations for treatment of asthma. The first combination comprises of indacaterol acetate and mometasone furoate (LABA/ICS), and another combination contains indacaterol acetate, glycopyrronium bromide and mometasone furoate (LABA/LAMA/ICS) [10, 17].

Findings from an experimentally informed biophysical modelling of the pulmonary drug delivery process supported the formulation and process development of indacaterol acetate batches for clinical development [18].

The aim of this clinical salt-bridging study was to compare the lung function, pharmacokinetics (PK), and safety of indacaterol maleate 150 μg o.d. and indacaterol acetate 150 μg o.d. versus placebo in patients with asthma.

## Methods

### Study design

This was a double-blind, placebo-controlled, three-period complete block, cross-over, US-based multicenter Phase II study in patients with asthma (ClinicalTrials.gov identifier, NCT03257995).

**Fig. 1:**
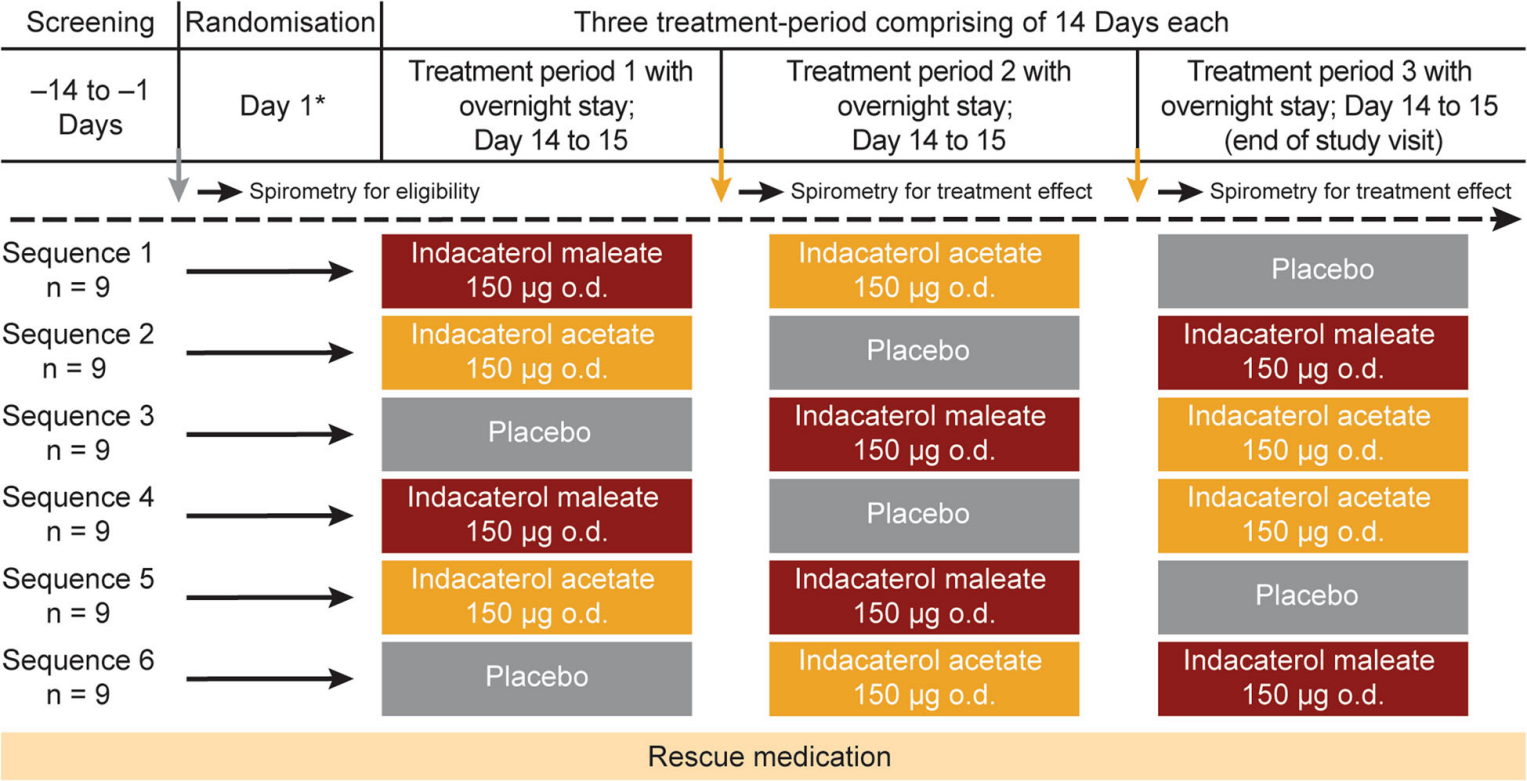
Study design. *o.d, once daily

The study was conducted between September 5, 2017 and January 18, 2018 and included a 14-day screening period to assess the eligibility of patients. The study comprised three treatment periods with a washout period of 7–14 days in between. Patients were randomised (1:1:1:1:1:1) to one of six treatment sequences across three different treatment periods (14 days each) to receive indacaterol maleate (150 μg o.d.), indacaterol acetate (150 μg o.d.) or placebo (Fig. 1). All treatments were delivered using the Breezhaler® inhaler. Patients were provided with an inhaled short-acting β_2_-agonist (SABA; 100 μg/puff salbutamol or other SABA) at matching dose-strength as rescue medication throughout the study and remained on background ICS at the dose strength taken during screening.

### Patients

Male and female patients (aged ≥18 years) with asthma (physician documented) for > 1 year, receiving a stable dose of ICS for ≥4 weeks prior to screening were included in the study if they had a pre-bronchodilator FEV_1_ ≥ 50% and ≤ 90% of the predicted normal value at screening, and demonstrated an increase in FEV_1_ of ≥12% and ≥ 200 mL within 30 min after administration of 400 μg salbutamol/360 μg albuterol (or equivalent dose) at screening. Patients were excluded from the study if they had experienced an asthma attack/exacerbation requiring systemic corticosteroids or hospitalisation or emergency room visit 6 weeks prior to screening, were current smokers (patients who have smoked or inhaled tobacco products 6 months prior to screening, or who have a smoking history of greater than 10 pack years), had previous intubation for a severe asthma attack/exacerbation, required the use of ≥12 puffs/24 h of rescue medication for 48 h (over 2 consecutive days) during screening prior to randomisation (list of key inclusion and exclusion criteria is provided in the supplementary material).

### Objectives and assessments

#### Lung function

The primary objective of the study was to assess the bronchodilator effect of indacaterol acetate and indacaterol maleate salts compared with placebo in terms of trough FEV_1_ (mean of FEV_1_ at 23 h 15 min and 23 h 45 min) after 14 days of treatment.

As secondary objectives, we evaluated the standardized FEV_1_ area under the curve from 0 to 4 h (FEV_1_ AUC_0–4h_, as well as FEV_1_, forced vital capacity (FVC) and forced expiratory flow (FEF_25–75%_) at each post-dose time point after 14 days of treatment. The spirometry measurements were performed during the following time points: 45 min and − 15 min, 5 min, 15 min, 30 min,1 h, 2 h, 4 h, 8 h, 12 h, 23 h 15 min, 23 h 45 on Day 14. Peak expiratory flow (PEF) rate was assessed b.i.d., once in the morning and once in the evening (~ 12 h later) throughout the study PEF measurements were taken using the peak flow meter device taken between Day 8 and 14 days of each treatment were pre-specified to be analysed as a secondary objective.

### Rescue medication

The use of rescue medication was recorded throughout the entire study taken b.i.d. using the eDiary and compared between the indacaterol salts and placebo on Days 8 and 14.

### Pharmacokinetic endpoints

Steady-state PK, including area under the plasma concentration vs. time curve from 0 to 24 h (AUC_0-24h,ss_), maximum (C_max,ss_), minimum (C_min,ss_), and average (C_avg,ss_) plasma concentration and time to reach maximal concentrations (T_max,ss_) were also assessed as secondary objectives.

### Safety

Safety assessments included monitoring of all adverse events (AEs) and serious adverse events (SAEs), evaluating their severity and relationship to treatment.

### Pharmacokinetic analysis

Blood samples for PK analysis on Day 14 in each period were collected at pre-dose, 0.08, 0.16, 0.25, 0.50, 1, 2, 4, 8, 12 and 24 h post-dose. All blood samples were taken by either direct venipuncture or an indwelling catheter inserted in a forearm vein. At specified time points, 2 mL blood sample was collected in lithium heparin tubes. Within 15 min, the sample was centrifuged at 4 °C for 15 min at approximately 1500 g. All plasma samples were frozen within 30 min of collection and stored at − 20 °C or colder, pending analyses. Samples corresponding to treatment periods where patients received placebo were not analysed. The concentrations of indacaterol in plasma were determined by a validated liquid chromatography–mass spectrometry/ mass spectrometry (LC-MS/MS) method [19]; the Lower Limit of Quantification (LLOQ) was 5.00 pg/mL. Concentrations were expressed in pg/mL units and referred to the free base of indacaterol. Concentrations below the LLOQ were treated as zero in summary statistics of concentration data as well as PK parameter calculations. PK parameters (AUC_0-24h,ss_, C_max,ss_, T_max,ss_) were determined using WinNonlin Phoenix (version 6.4; Certara, Princeton, NJ, USA).

### Statistical analysis

The PK/PD analysis sets included all patients who received any study drug and experienced no major protocol deviations with relevant impact on PK/PD data. Sample size calculations were based on the primary endpoint of trough FEV_1_, assuming within-patient standard deviation (SD) of 230 mL (derived from an earlier study, data on file) and with 42 patients completing the study, the power to detect a significant difference between indacaterol salts and placebo at the 2-sided 5% alpha level, assuming a true difference of at least 170 mL in trough FEV_1_, would be at least 90%. The primary endpoint, trough FEV_1_, after 14 days of treatment was analysed using Analysis Of Variance (ANOVA) with treatment, period and sequence as fixed effects and subject within sequence as a random effect.

Standardised FEV_1_ AUC_0-4h_ between baseline (pre-dose) and 4 h post-dose on Day 14 was analysed using the same ANOVA model as for the primary. The remaining secondary spirometry parameters were analysed using repeated measures analysis of variance (ANOVA) model, with treatment, period, and sequence as fixed effects and subject nested within sequence was included as random effect. Time was repeated within each subject by period interaction term. An unstructured variance-covariance matrix was considered for the repeated measures residuals. The adjusted mean difference between indacaterol salts and placebo and their corresponding two-sided 95% confidence intervals (CIs) along with *P* values for the differences were reported. The log-transformed PK parameters AUC_0-24h,ss_ and C_max,ss_ on Day 14 were compared for indacaterol acetate (test) relative to indacaterol maleate (reference) using a mixed-effects model with sequence, treatment, and period as fixed effects and patient nested within sequence as random effect. T_max,ss_ was analysed using non-parametric methods. The median difference and the 90% CI of the median difference in T_max,ss_ was estimated using Hodges-Lehmann estimation procedure. Averaged morning and evening puffs and overall number of puffs of rescue medications and averaged morning and evening and overall number of PEF measurements were also analysed using ANOVA similar to trough FEV_1_.

The safety summaries included all patients who received any treatment (indacaterol salts and/or placebo). AEs by system organ class (SOC) and preferred term (PT), and AEs leading to permanent discontinuation of study drug by SOC and PT with a breakdown by treatment subject with multiple AEs within a body system and treatment period, were only counted once toward the total of the body system and treatment.

## Results

### Patients

In total, 54 patients were randomised, with nine patients in each treatment sequence. Of these, 51 completed the study. Three patients discontinued the study; one due to AE (upper respiratory tract infection and asthma exacerbation) and two discontinued due to deviations from study protocol (administration of prohibited concomitant medication). The median age of the patients in the study was 48 years (range: 26–70). Most patients were female (66.7%). The population mean ± SD body mass index was 29.9 ± 5.1 kg/m^2^ (Table 1). Patients had mean pre-bronchodilator FEV_1_ of 2.229 L and mean post-bronchodilator FEV_1_ of 2.693 L with an average reversibility of 21% at baseline.

**Table 1:** Demographic and clinical characteristics at baseline

Parameters	Total (***N*** = 54)
Age, years, median (range)	48 (26–70)
Sex, n (%)	
Men	18 (33.3)
Women	36 (66.7)
Body mass index (kg/m^2^)	29.9 ± 5.1
Race, n (%)	Black 7 (13.0)
	White 47 (87.0)
Ethnicity, n (%)	Hispanic or Latino 8 (14.8)
	Not Reported 30 (55.6)
	Unknown 8 (14.8)
	Other 8 (14.8)
Pre-bronchodilator FEV_1_, L	2.229 ± 0.679
Post-bronchodilator FEV_1_, L	2.693 ± 0.816
Pre-bronchodilator FEV_1_, % predicted	71 ± 11
Post-bronchodilator FEV_1_, % predicted	86 ± 12
FEV_1_ Reversibility^a^, L	0.464 ± 0.241
FEV_1_ Reversibility^b^ (%)	21 ± 10

Data are represented as mean ± SD unless otherwise specified

*FEV*_*1*_ Forced expiratory volume in 1 s, *SD* Standard deviation

^a^ Reversibility (L) is calculated as: FEV_1_ (post-bronchodilator) - FEV_1_ (pre-bronchodilator)

^b^ Reversibility (%) is calculated as: (FEV_1_ (post-bronchodilator) - FEV_1_ (prebronchodilator) × 100)/ FEV_1_ (prebronchodilator)

### Lung function

After 14 days of treatment, both indacaterol maleate (*N* = 47) and indacaterol acetate (*N* = 49) showed a statistically significant improvement in trough FEV_1_ compared with placebo (both *P* < 0.001).

**Fig. 2:**
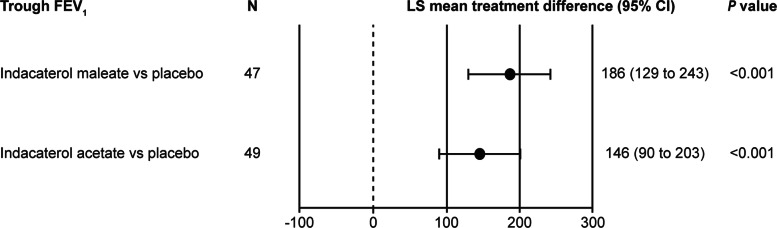
Significant improvement in trough FEV_1_ (mL) with indacaterol maleate and indacaterol acetate versus placebo at Day 14. Data presented as LS mean treatment differences (95% CI). CI, confidence interval; FEV_1_, forced expiratory volume in 1 s; LS, least square; N, number of patients

The LS mean treatment differences versus placebo in trough FEV_1_ with indacaterol maleate was 186 mL (95% CI, 129 to 243 mL) and with indacaterol acetate was 146 mL (95% CI, 90 to 203 mL) (*P* < 0.001 for both comparisons; Fig. 2).

**Fig. 3:**
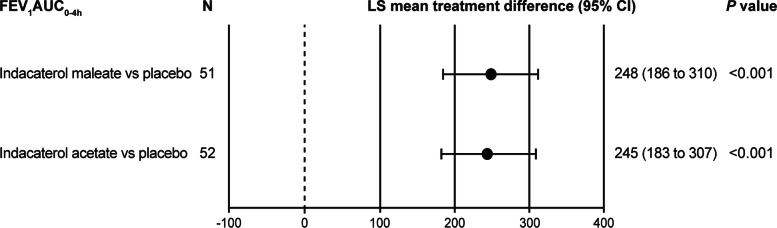
Improvement in FEV_1_ AUC_0-4h_ (mL) with indacaterol maleate and indacaterol acetate versus placebo at Day 14. Data presented as LS mean treatment differences (95% CI). AUC, area under the curve; CI, confidence interval; FEV_1_, forced expiratory volume in 1 s; LS, least square; N, number of patients

After 14 days of treatment the LS mean treatment differences versus placebo in FEV_1_ AUC_0-4h_ with indacaterol maleate (*N* = 51) was 248 mL, and 245 mL with indacaterol acetate (*N* = 52; *P* < 0.001 for both comparisons; Fig. 3).

**Fig. 4:**
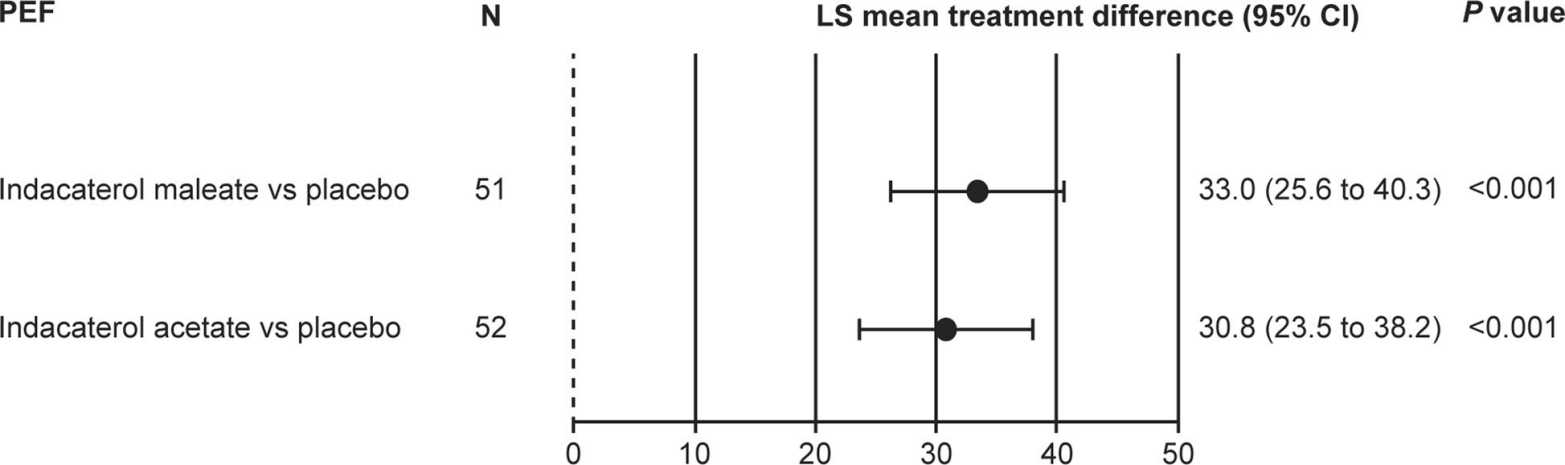
Improvement in PEF (L/min) with indacaterol maleate and indacaterol acetate versus placebo at Day 14. Data are presented as LS mean treatment difference (95% CI). LS, least square; N, No. of patients; PEF, peak expiratory flow

Indacaterol maleate and indacaterol acetate showed substantial improvements in the mean pre-dose morning, evening, and overall PEF compared with placebo over Days 8–14 of each respective treatment period. The mean treatment difference in morning PEF compared with placebo was 30.9 L/min and 29.6 L/min for indacaterol maleate (*N* = 51) and indacaterol acetate (*N* = 52), respectively, and the mean treatment difference in evening PEF compared with placebo was 34.8 L/min and 32.7 L/min for indacaterol maleate and indacaterol acetate respectively. Improvements were observed in overall PEF for both the indacaterol salts compared with placebo (overall PEF; Fig. 4). Both indacaterol salts (maleate and acetate) showed comparable and statistically significant improvements in other assessed lung function parameters at all assessed time points compared with placebo (FEV_1_: Table S1, FVC: Table S2, FEF_25–75%_: Table S3).

#### Rescue medication

The LS mean for rescue medication use between Days 8–14 of each treatment period was 1.01 puffs/day each for both the indacaterol salts (maleate, *N* = 51; acetate, *N* = 52) and 1.43 puffs/day for placebo (*N* = 51). A reduction of 0.42 in LS mean puffs/day was observed with both indacaterol salts compared with placebo (maleate, *P* = 0.009; acetate, *P* = 0.008).

#### Systemic pharmacokinetics

**Fig. 5:**
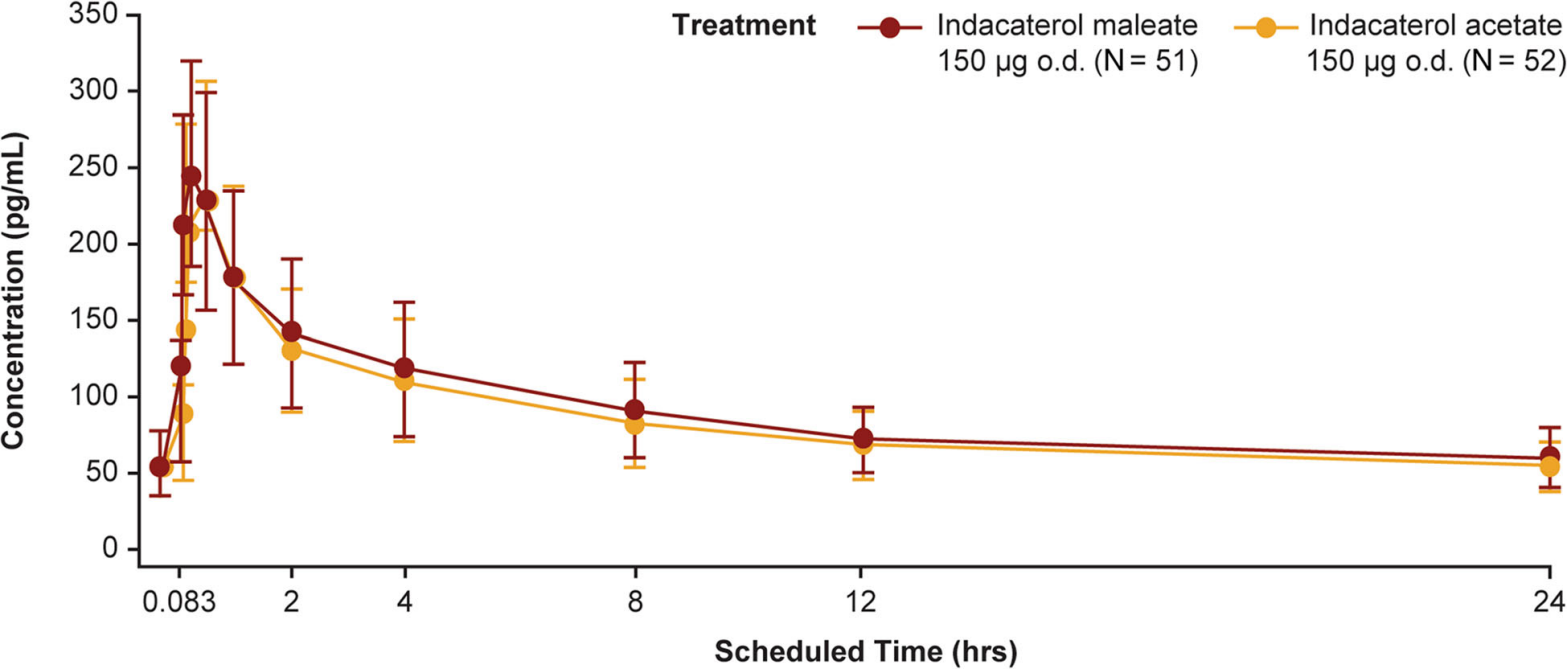
Plasma concentration–time profiles for indacaterol maleate and indacaterol acetate on Day 14. Data presented as arithmetic mean ± SD; error bars indicate SD values; o.d., once daily

Indacaterol maleate and indacaterol acetate showed similar systemic plasma concentration–time profiles on Day 14 (Fig. 5). Both indacaterol salts showed rapid absorption with peak concentrations achieved within 30 min post-inhalation.

There was no relevant difference in exposure (AUC_0–24h_,_ss_ and C_max,ss_) at steady state between indacaterol acetate and indacaterol maleate (Table 2). The median time to reach peak concentration (T_max_) post-dose was 15 min with indacaterol maleate and 28 min with indacaterol acetate.

**Table 2:** Summary statistics of plasma PK parameters for indacaterol salts on Day 14

	Indacaterol maleate 150 μg o.d. (***N*** = 51)	Indacaterol acetate 150 μg o.d. (***N*** = 52)
AUC_0-24h,ss_, h*pg/mL	2300 ± 732 (31.8)	2050 ± 636 (31.0)
C_max,ss_, pg/mL	264 ± 80.2 (30.3)	236 ± 74.0 (31.3)
T_max,ss,_ h	0.250 (0.18 to 0.85)	0.467 (0.18 to 1.00)
C_min,ss_ (pg/mL)	56.4 ± 21.7 (38.4)	52.6 ± 18.1 (34.4)
C_av,ss_ (pg/mL)	94.1 ± 31.3 (33.3)	85.5 ± 26.5 (31.0)

All values are represented as mean ± SD (CV%), except for T_max,ss_ which is presented as median (range)

*AUC* Area under the curve, *C*_*av*_ Average plasma concentration, *C*_*max*_ Maximum plasma concentration, *C*_*min*_ Minimum plasma concentration, *CV* Coefficient of variation, *o.d.* Once daily, *PK* Pharmacokinetics, *SD* Standard deviation, *T*_*max*_ Time to reach maximal concentrations

Geometric mean ratios (indacaterol acetate vs. indacaterol maleate) and the corresponding 90% CIs for both AUC_0-24h_,_ss_ and C_max,ss_ were within the bioequivalence limits (90% CI range: 0.80 to 1.25) [20], indicating a comparable exposure from both the indacaterol salts (Table 3).

**Table 3:** Comparative analysis of PK between indacaterol maleate and acetate on Day 14

	Indacaterol maleate 150 μg o.d. (***N*** = 51)	Indacaterol acetate 150 μg o.d. (***N*** = 52)	Indacaterol acetate versus Indacaterol maleate
Geometric LSM ratio (90% CI)			
AUC_0-24h,ss_, h*pg/mL	2180 (2020 to 2350)	1950 (1820 to 2100)	0.897 (0.854 to 0.942)
C_max,ss_, pg/mL	253 (236 to 273)	226 (210 to 243)	0.891 (0.846 to 0.939)

*AUC* Area under the curve, *C*_*max*_ Maximum plasma concentration, *LSM* Least square mean, *o.d.* Once daily, *PK* Pharmacokinetics

#### Safety

Safety data for all study treatments is shown in Table 4. The overall incidence of AEs was 27.5% for indacaterol maleate, 13.5% for indacaterol acetate, and 17.0% for placebo. Indacaterol acetate was not associated with AEs of cough (*n* = 0), whereas 12 patients (23.5%) reported cough with indacaterol maleate. Four severe AEs were reported in two patients after they received indacaterol acetate 150 μg (3.8%, *n* = 2), one patient reported streptococcal pharyngitis and sinusitis and the other patient reported upper respiratory tract infection and asthma exacerbation as severe AE. one severe AE (cholangitis) was reported in a patient treated with placebo (1.9%, *n* = 1). In total, two serious adverse events (SAEs), pharyngitis streptococcal and sinusitis were reported in one patient during the indacaterol acetate treatment period. This patient discontinued from the study; overall, none of the severe AEs or SAEs were considered to be related to the study treatments by the investigator.

**Table 4:** Incidence of treatment-emergent AEs by preferred term affecting ≥3% of total patients (safety analysis set)

	Indacaterol maleate 150 μg o.d. ***n*** = 51 n (%)	Indacaterol acetate 150 μg o.d. ***n*** = 52 n (%)	Placebo ***n*** = 53 n (%)	Total ***N*** = 54 n (%)
Number of patients with ≥1 AE	14 (27.5)	7 (13.5)	9 (17.0)	24 (44.4)
Cough	12 (23.5)	0 (0.0)	1 (1.9)	13 (24.1)
Upper respiratory tract infection	1 (2.0)	2 (3.8)	2 (3.8)	5 (9.3)
Arthralgia	0 (0.0)	1 (1.9)	1 (1.9)	2 (3.7)
Sinusitis	0 (0.0)	1 (1.9)	1 (1.9)	2 (3.7)
Vomiting	1 (2.0)	1 (1.9)	0 (0.0)	2 (3.7)

Data presented as n (%)

*AE* Adverse event, *o.d.* Once daily

## Discussion

The findings from this Phase II salt-bridging study demonstrate the efficacy and safety of indacaterol maleate and indacaterol acetate in patients with asthma. It also provides evidence of similar systemic exposure to indacaterol following administration of either of its salt forms.

The primary objective was met with both indacaterol salts (maleate and acetate) demonstrating statistically significant (*P* < 0.001) and clinically meaningful improvements in mean trough FEV_1_ compared with placebo (186 mL and 146 mL, respectively) following the 14-day treatment period on top of stable ICS background therapy. These improvements are within the range reported in studies that investigated other LABAs such as formoterol and salmeterol b.i.d. in patients with asthma [21–24], although these studies assessed change in FEV_1_ at different time points from the present study.

Donohue et al. observed that indacaterol o.d. showed similar improvements in trough FEV_1_ at Day 14 and Day 84 [25]. Improvements in lung function were also observed in secondary endpoints including FEV_1_, AUC_0-4h_, PEF, and FVC. These findings support the substantial bronchodilator effect of indacaterol and subsequent improvement in airflow limitation, which is one of the key goals for patients with asthma [1].

Upon comparison of indacaterol acetate vs. indacaterol maleate, the geometric mean ratio (90% CI) for AUC_0-24h,ss_ was 0.897 (90% CI: 0.854, 0.942) and for C_max,ss_ was 0.891 (90% CI: 0.846, 0.939). Thus, the geometric mean ratio and 90% CI for both AUC_0-24h,ss_ and C_max_ fell well within the bioequivalence limits (90% CI: 0.80 to 1.25) [20], indicating similar exposure from both salts. The PK parameters (AUC_0-24h,ss_, C_max,ss_ and T_max,ss_) observed for indacaterol in this study were comparable to those reported previously following administration of 150 μg indacaterol acetate or indacaterol maleate at steady state in healthy volunteers [26, 27].

Although formulation bridging studies are typically conducted as single dose crossover studies in healthy volunteers, the use of a multiple dose crossover study design in asthma patients in the present study allowed evaluation of both PK and PD parameters for both salt forms in the intended indication at steady state.

Systemic PK following inhaled administration depends on a number of unique factors including the delivered dose (dose ex-mouth piece), fine particle mass or the respirable fraction of an inhaled dose, performance of the inhalation device and the ability of subjects to correctly use the inhalation device. Use of an experimentally informed biophysical modeling of the pulmonary drug delivery process to support the formulation and process development of indacaterol acetate batches for clinical development was a critical factor that enabled a successful bridging program for the indacaterol salts.

In the present study, patients treated with indacaterol maleate and indacaterol acetate demonstrated considerable reduction in the use of rescue medication compared with placebo. These improvements support the therapeutic benefit of indacaterol in controlling asthma symptoms. In addition, lowered use of rescue medication with indacaterol may help to alleviate side effects like cataract, diabetes, peptic ulcers, cardiovascular disease (CVD), cerebrovascular events and osteoporosis that are generally associated with the frequent and long term use of reliever medications [28–30].

Overall, the safety profile of both indacaterol salt forms was comparable with placebo and consistent with the established safety profile for inhaled indacaterol [31, 32]. The only notable difference was that indacaterol acetate was not associated with AEs of coughing in any of the patients, whereas it was observed in 12 patients in the indacaterol maleate treatment arm and in one patient in the placebo arm. No new safety signals were observed compared to those seen in previous studies with indacaterol [24, 31, 33]. The two SAEs of streptococcal pharyngitis and sinusitis were not suspected to be related to the study drug by the investigator.

A washout period of 7–14 days eliminated any potential carryover effect from the previous treatment period. Within-patient variability in FEV_1_ was predicted to be less than between-patient variability. Taking this into consideration, the crossover study design was chosen over a parallel-group design. The 14-day treatment period selected was sufficient to assess PK parameters at steady-state and to assess the effect of indacaterol treatment on lung function. It is supported by the observable change reported in previous studies [34, 35], which showed that indacaterol achieved PD steady-state within 14 days of once-daily administration in COPD patients [34, 35].

Indacaterol acetate was comparable to indacaterol maleate (both at a dose of 150 μg o.d.) in terms of efficacy, systemic exposure and safety. These findings support the utility of indacaterol acetate as a LABA in two currently developed inhaled fixed dose combination therapies for asthma: a once-daily LABA/ICS combination with mometasone furoate, and a once-daily LABA/LAMA/ICS combination with glycopyrronium bromide and mometasone furoate.

## Conclusions

In patients with asthma, both indacaterol maleate and indacaterol acetate achieved significant improvements in lung function compared with placebo and elicited similar systemic exposure. Both indacaterol salts were safe and overall well tolerated; no AEs of cough were observed with indacaterol acetate. These findings support indacaterol acetate as a potent LABA in inhaled combination therapies for patients with asthma.

## Supplementary information

**Additional file 1.**

## Data Availability

Novartis is committed to sharing with qualified external researchers, access to patient-level data and supporting clinical documents from eligible studies. These requests are reviewed and approved by an independent review panel on the basis of scientific merit. All data provided are anonymized to respect the privacy of patients who have participated in the trial in line with applicable laws and regulations.

## Abbreviations

ANOVA: Analysis Of Variance
AEs: Adverse events
ECG: Electrocardiograms
FDC: Fixed dose combination
FEV_1_: Forced expiratory volume in 1 s
FEV_1_ AUC: Forced expiratory volume in 1 s area under the curve
FVC: Forced vital capacity
ICS: Inhaled corticosteroid
LABA: Long-acting β_2_-agonist
LABA/ICS: Long-acting β_2_-agonist/inhaled corticosteroid
LABA/LAMA/ICS: Long-acting β_2_-agonist/long-acting muscarinic antagonist/inhaled corticosteroids
LABA/LAMA: Long-acting β_2_-agonist/long-acting muscarinic antagonist
LAMA: Long-acting muscarinic-antagonist
LC-MS/MS: Liquid chromatography–mass spectrometry/ mass spectrometry
PK: Pharmacokinetics
PD: Pharmacodynamics
SAEs: Serious adverse events
