# Supplementary material for: Lung function, pharmacokinetics, and tolerability of inhaled indacaterol maleate and acetate in asthma patients
Source: Respir Res. 2020 Sep 23;21:248. doi: 10.1186/s12931-020-01501-1 (PMC7513528; doi:10.1186/s12931-020-01501-1)
Supplement: Supplementary file 1 — Additional file 1. [file 12931_2020_1501_MOESM1_ESM.docx]

**Supplementary information**

**Lung function, pharmacokinetics, and tolerability of inhaled indacaterol maleate and acetate in asthma patients**

David Miller^1^, Soniya Vaidya^2*^, Juergen Jauernig^3^, Brian Ethell^4^, Kristina Wagner^4^, Rajkumar Radhakrishnan^5^, Hanns-Christian Tillmann^6**^

**^1^Northeast Medical Research Associates Inc at North Dartmouth, MA, United States; ^2^Axcella Health, Cambridge, MA, United States; ^3^Novartis Pharma AG, Basel, Switzerland;
^4^Novartis Institutes for Biomedical Research, Cambridge, MA, United States; ^5^Novartis Healthcare Pvt. Ltd., Hyderabad, India; ^6^Novartis Institutes for Biomedical Research, Translational Medicine, Basel, Switzerland**

**Key Inclusion Criteria:**

- Male and female patients aged ≥ 18 years and above
- Patients with a documented physician diagnosis of asthma for a period of at least 1 year prior to screening and who additionally meet the following criteria:
  - Patients receiving daily treatment with an inhaled corticosteroid up to the maximum dose per day (as indicated in the package leaflet), on a stable regimen for at least 4 weeks prior to screening.
- Pre-bronchodilator forced expiratory volume in one second (FEV_1_) ≥50 % and ≤90% of the predicted normal value for the patient during screening.
- Patients who demonstrate an increase in FEV_1_ of ≥ 12% and ≥ 200 mL after administration of 400 μg salbutamol/360 μg albuterol (or equivalent dose) at screening.
- Subjects must weigh at least 50 kg at screening to participate in the study, and must have a body mass index (BMI) within the range of 18 to 40 kg/m^2^

**Key Exclusion Criteria:**

- Patients who have had an asthma attack/exacerbation requiring systemic steroids or hospitalization or emergency room visit within 6 weeks of screening.
- Patients who have had a respiratory tract infection or asthma worsening within 4 weeks prior to screening.
- Patients with a history of chronic lung diseases other than asthma
- Patients who have a decline in PEF from the reference PEF (taken at screening) of ≥30% for 5 of 6 consecutive scheduled PEF readings (readings taken at morning and evening) during at least 3 days of screening epoch prior to randomization.
- Patients who require the use of ≥12 puffs / 24 hours of rescue medication for 48 hours (over two consecutive days) during screening prior to randomization.
- Pregnant or nursing (lactating) women, where pregnancy is defined as the state of a female after conception and until the termination of gestation, confirmed by a positive hCG laboratory test.
- Patients with Type I diabetes or uncontrolled Type II diabetes (HbA1c > 9%) at screening.
- Current smokers (urine cotinine > than the laboratory's lowest level of quantification (LoQ of 500 ng/mL or lower)) and patients who have smoked or inhaled tobacco products within the 6 month period prior to screening, or who have a smoking history of greater than 10 pack years (Note: 1 pack is equivalent to 20 cigarettes. 10 pack years = 1 pack /day x 10 yrs., or ½ pack/day x 20 yrs.).
- Contraindicated for treatment with, or having a history of reactions/ hypersensitivity to any of the following inhaled drugs, drugs of a similar class, or any component thereof: Sympathomimetic amines / adrenoceptor agonist agents Lactose or any of the other excipients of the study drug (including patients with history of galactose intolerance, Lapp lactase deficiency or glucose-galactose malabsorption)

**Table S1: Improvement in FEV_1_ (L) in patients treated with indacaterol acetate and indacaterol maleate compared with placebo on Day 14**

| **Indacaterol acetate versus placebo** | | | **Indacaterol maleate versus placebo** | | |
| --- | --- | --- | --- | --- | --- |
| Time point (h) | LS mean treatment difference | 95 % CI; (*P*-value) | Time point (h) | LS mean treatment difference | 95% CI; *P*-value |
| 5 min | 0.2195 | 0.1502 to 0.2889; <0.001 | 5 min | 0.2177 | 0.1482 to 0.2872; <0.001 |
| 15 min | 0.2684 | 0.1988 to 0.3380; <0.001 | 15 min | 0.2724 | 0.2030 to 0.3417; <0.001 |
| 30 min | 0.2572 | 0.1897 to 0.3266; <0.001 | 30 min | 0.2730 | 0.2036 to 0.3423; <0.001 |
| 1 h | 0.2348 | 0.1656 to 0.3040; <0.001 | 1 h | 0.2609 | 0.1915 to 0.3302; <0.001 |
| 2 h | 0.2546 | 0.1852 to 0.3239; <0.001 | 2 h | 0.2494 | 0.1800 to 0.3188; <0.001 |
| 4 h | 0.2322 | 0.1627 to 0.3017; <0.001 | 4 h | 0.2273 | 0.1578 to 0.2968; <0.001 |
| 8 h | 0.2324 | 0.1627 to 0.3020; <0.001 | 8 h | 0.2396 | 0.1697 to 0.3096; <0.001 |
| 12 h | 0.2057 | 0.1359 to 0.2755; <0.001 | 12 h | 0.2443 | 0.1742 to 0.3144; <0.001 |
| 23 h 15 min | 0.1625 | 0.0912 to 0.2337; <0.001 | 23 h 15 min | 0.2280 | 0.1563 to 0.2997; <0.001 |
| 23 h 45 min | 0.1793 | 0.1080 to 0.2505; <0.001 | 23 h 45 min | 0.1954 | 0.1237 to 0.2671; <0.001 |
| LS, least square; FEV_1_, forced expiratory volume in one second | | | | | |

**Table S2: Improvement in FVC in patients treated with indacaterol acetate and indacaterol maleate compared with placebo on Day 14**

| **Indacaterol acetate versus placebo** | | | **Indacaterol maleate versus placebo** | | |
| --- | --- | --- | --- | --- | --- |
| Time point (h) | LS mean treatment difference | 95 % CI; (*P-*value) | Time point (h) | LS mean treatment difference | 95 % CI; (*P*-value) |
| 5 min | 0.2130 | 0.1386 to 0.2873; <0.001 | 5 min | 0.1866 | 0.1121 to 0.2611; <0.001 |
| 15 min | 0.2173 | 0.1426 to 0.2920; <0.001 | 15 min | 0.2249 | 0.1506 to 0.2992; <0.001 |
| 30 min | 0.2375 | 0.1632 to 0.3118; <0.001 | 30 min | 0.2404 | 0.1661 to 0.3148; <0.001 |
| 1 h | 0.2013 | 0.1272 to 0.2754; <0.001 | 1 h | 0.2074 | 0.1330 to 0.2817; <0.001 |
| 2 h | 0.2005 | 0.1262 to 0.2749; <0.001 | 2 h | 0.1802 | 0.1059 to 0.2545; <0.001 |
| 4 h | 0.1946 | 0.1201 to 0.2691; <0.001 | 4 h | 0.1621 | 0.0876 to 0.2366; <0.001 |
| 8 h | 0.1937 | 0.1190 to 0.2684; <0.001 | 8 h | 0.1990 | 0.1239 to 0.2742; <0.001 |
| 12 h | 0.1619 | 0.0870 to 0.2369; <0.001 | 12 h | 0.1747 | 0.0994 to 0.2501; <0.001 |
| 23 h 15 min | 0.1249 | 0.0480 to 0.2018; <0.001 | 23 h 15 min | 0.1856 | 0.1081 to 0.2631; <0.001 |
| 23 h 45 min | 0.1546 | 0.0777 to 0.2315; <0.001 | 23 h 45 min | 0.1484 | 0.0709 to 0.2259; <0.001 |
| LS, least square; FVC, Forced expiratory flow at 25% to 75% of forced vital capacity | | | | | |

**Table S3: Improvement in FEF_25-75%_ in patients treated with indacaterol acetate and indacaterol maleate compared with placebo on Day 14**

| **Indacaterol acetate versus placebo** | | | **Indacaterol maleate versus placebo** | | |
| --- | --- | --- | --- | --- | --- |
| Time point (h) | LS mean treatment difference | 95 % CI; (*P*-value) | Time point (h) | LS mean treatment difference | 95 % CI; (*P*-value) |
| 5 min | 0.1956 | 0.1012 to 0.2899; < 0.001 | 5 min | 0.029 | 0.1059 to 0.2952; <0.001 |
| 15 min | 0.2927 | 0.1979 to 0.3875; <0.001 | 15 min | 0.0330 | 0.1979 to 0.3867; <0.001 |
| 30 min | 0.2723 | 0.1780 to 0.3667; <0.001 | 30 min | 0.0306 | 0.1780 to 0.3667; <0.001 |
| 1 h | 0.2386 | 0.1272 to 0.2754; <0.001 | 1 h | 0.0324 | 0.1445 to 0.3328; <0.001 |
| 2 h | 0.2986 | 0.1262 to 0.2749; <0.001 | 2 h | 0.0338 | 0.1875 to 0.3763; <0.001 |
| 4 h | 0.2661 | 0.1201 to 0.2691; <0.001 | 4 h | 0.0325 | 0.1874 to 0.3766; <0.001 |
| 8 h | 0.2558 | 0.1190 to 0.2684; <0.001 | 8 h | 0.0311 | 0.1585 to 0.3492; <0.001 |
| 12 h | 0.1987 | 0.0870 to 0.2369; <0.001 | 12 h | 0.0359 | 0.1732 to 0.3643; <0.001 |
| 23 h 15 min | 0.2044 | 0.0480 to 0.2018; <0.001 | 23 h 15 min | 0.0326 | 0.1473 to 0.3434; <0.001 |
| 23 h 45 min | 0.1997 | 0.0777 to 0.2315; <0.001 | 23 h 45 min | 0.0301 | 0.0882 to 0.2842; <0.001 |
| LS, least square; FEF, forced expiratory flow | | | | | |
